# Respiratory chain deficiency in aged spinal motor neurons^☆^

**DOI:** 10.1016/j.neurobiolaging.2014.02.027

**Published:** 2014-10

**Authors:** Karolina A. Rygiel, John P. Grady, Doug M. Turnbull

**Affiliations:** a Newcastle University Centre for Brain Ageing and Vitality, Institute for Ageing and Health, The Medical School, Newcastle University, Newcastle upon Tyne, UK; b Wellcome Trust Centre for Mitochondrial Research, Institute for Ageing and Health, The Medical School, Newcastle University, Newcastle upon Tyne, UK

**Keywords:** Sarcopenia, Spinal cord, Motor neuron, Mitochondria, mtDNA, Complex I

## Abstract

Sarcopenia, muscle wasting, and strength decline with age, is an important cause of loss of mobility in the elderly individuals. The underlying mechanisms are uncertain but likely to involve defects of motor nerve, neuromuscular junction, and muscle. Loss of motor neurons with age and subsequent denervation of skeletal muscle has been recognized as one of the contributing factors. This study investigated aspects of mitochondrial biology in spinal motor neurons from elderly subjects. We found that protein components of complex I of mitochondrial respiratory chain were reduced or absent in a proportion of aged motor neurons–a phenomenon not observed in fetal tissue. Further investigation showed that complex I-deficient cells had reduced mitochondrial DNA content and smaller soma size. We propose that mitochondrial dysfunction in these motor neurons could lead to the cell loss and ultimately denervation of muscle fibers.

## 1 Introduction

Sarcopenia is an age-related process with loss of muscle mass and strength decline. Proposed diagnostic criteria for sarcopenia include a gait speed of less than 1 m/s and an objectively low muscle mass (typically 2 standard deviations below the mean muscle mass of 35-year-old individual) (Fielding et al., 2011). Between the ages of 20 and 80 years there is approximately a 30% reduction in muscle mass, which is reflected in markedly reduced cross-sectional area of muscle (about 20%) (Frontera et al., 2000). It has been estimated that up to 20% of people between 60 and 70 years are affected by sarcopenia, and this number increases to 50% for those aged 80 or above (von Haehling et al., 2010).

The mechanisms driving sarcopenia are uncertain but they are likely to be multifactorial, with involvement of both muscle and the innervating neurons (Delbono, 2003). There is good evidence of denervation with fiber type grouping, accumulation of severely atrophic angular fibers, and expression of proteins associated with denervation such as NCAM and Nav1.5 by the atrophic fibers (Andersen, 2003; Lexell and Downham, 1991; Lexell and Taylor, 1991; Urbanchek et al., 2001; Wang et al., 2005). Fibers co-expressing myosin heavy chain slow and fast isoforms with Nav1.5 sodium channel were detected in aged rodent gastrocnemius (Rowan et al., 2012), also indicating presence of denervation as an important factor in sarcopenia across species.

Neurophysiological assessments in both animals and humans provide further evidence of denervation of muscle with age. Functional motor unit numbers were shown to decline with age and the remaining motor units became larger (Stalberg and Fawcett, 1982). In humans, a marked reduction in motor units of up to 30% occurs between the age of 60 and 70 years (Brown et al., 1988; Campbell et al., 1973; Doherty and Brown, 1993). Interestingly, motor unit reduction is not immediately followed by decline in muscle strength. It has been shown in selected muscles that a noticeable decline in strength only occurs after a certain critical number of motor units have been lost (McNeil et al., 2005).

Depletion of motor units is most likely caused by reduction of the population of motor neurons residing in anterior spinal cord and innervating muscle fibers. Studies of lumbar spinal cord regions from healthy human subjects ranging from 13 to 95 years have shown a dramatic decrease in motor neuron soma counts in ventral horns with advancing age (Lexell, 1997; Tomlinson and Irving, 1977). The mean decrease determined was around 25%, which compares well with another study carried out on senescent rats where a 27% reduction of motor neuron number was noted (Rowan et al., 2012). Decrease of motor neuron soma counts is followed by decline in axonal density in ventral lumbar spinal roots. A study by Kawamura et al. (1977) on healthy humans (17–81 year old) revealed that approximately 5% of axons were lost every 10 years between the second and 10th decade of life. Intriguingly, studies on motor neuron loss with age from animal models provide conflicting results. Some report decrease in motor unit numbers correlated with loss of axons from innervating nerve trunks (Caccia et al., 1979; Valdez et al., 2010), others postulate that motor neuron pool is maintained and it is the degeneration of the neuromuscular junction that causes denervation of muscle fibers in senescent animals (Chai et al., 2011; Larsson, 1995).

There is a wide range of potential contributors to motor neuronal cell death in aging such as calcium dyshomeostasis, reduced IGF-1 signaling, proinflammatory cytokines, and oxidative stress (Aagaard et al., 2010; Landfield et al., 1992). However, in light of mitochondrial dysfunction reported in aged muscle fibers (Aiken et al., 2002; McKiernan et al., 2009) we decided to investigate mitochondrial biology in aged motor neurons. A number of previous studies have detected a variety of mitochondrial abnormalities in both sporadic and familial form of motor neuron disease (MND = amyotrophic lateral sclerosis [ALS]) (Borthwick et al., 1999; Comi et al., 1998; Wiedemann et al., 2002) but we are not aware of any systematic exploration of mitochondrial defects in aged motor neurons, which could relate to neurogenic changes seen in sarcopenia.

In this study we investigated mitochondrial changes in motor neurons from a group of elderly subjects. We identified a population of motor neurons with mitochondrial respiratory complex I deficiency. We discovered that these cells had lower mitochondrial DNA (mtDNA) copy numbers when compared with respiratory-normal counterparts. Moreover, cell bodies of these complex I-deficient motor neurons were markedly smaller than the healthy motor neurons.

## 2 Methods

### 2.1 Individuals studied

Lumbar region of spinal cords obtained from autopsies from 14 elderly individuals (68–99 years of age) without any diagnosed muscle disease were used in this study. Characterization of the individual cases is shown in Table 1. No spinal cord samples were available from young individuals and therefore fetal spinal cords of 9–11 weeks post conception were used as a control for selected experiments. Brain sections from a patient with a mitochondrial polymerase γ mutation were also used to confirm the specificity of the methods used.

### 2.2 Cytochrome c oxidase and succinate dehydrogenase histochemistry

Frozen spinal cord tissue sections, obtained from 12 elderly individuals (cut at 10 μm, obtained from Newcastle Brain Tissue Resource and London Multiple Sclerosis Society Tissue Bank) were mounted onto superfrost glass slides (Thermo Fisher, UK) and dried for 1 hour at room temperature. Combined cytochrome c oxidase (COX) and succinate dehydrogenase (SDH) enzymatic activity assay was performed in frozen spinal cord tissue from all 12 cases as previously described (Old and Johnson, 1989).

### 2.3 Immunohistochemistry to detect components of mitochondrial respiratory chain in paraffin sections

Paraffin-embedded spinal cord sections (5 μm thick, obtained from Newcastle Brain Tissue Resource, London Multiple Sclerosis Society Tissue Bank and Newcastle Human Developmental Brain Resource) were incubated at 60 °C for 20 minutes, then dewaxed in 2 changes of Histo-Clear, rehydrated in a gradient of ethanol (100% → 100% → 95% → 70%) and washed in TBST. Heat-induced antigen retrieval in 1 mM ethylenediaminetetraacetic acid pH 8.3 buffer was carried out in a decloaker (MenaPath, A. Menarini Diagnostics, UK) at 125 °C for 1 minute. The sections were washed in TBST and endogenous peroxidase was quenched by incubating the sections in 3% H_2_O_2_ in TBST for 15 minutes. The sections were incubated in 5% BSA and/or TBST solution for 30 minutes to reduce potential nonspecific binding of primary antibodies. Primary antibodies were applied onto the sections: subunits of complex I (cI-19, cI-20), complex IV (COX-I), complex II (cII-70), and porin. An overnight incubation was carried out in a humidified chamber at 4 °C. The following day, the sections were washed and the polymer detection system was used to develop the signal (MenaPath, A. Menarini Diagnostics, UK) as described in the following. The sections were covered with Universal Probe and incubated for 30 minutes, then washed in TBST and overlaid with Polymer-HRP reagent for 30 minutes. Following TBST wash, the sections were incubated with a chromogen solution: either DAB (Sigma Fast tablets, Sigma, UK) or Vector SG (Vector Laboratories, UK) prepared according to the manufacturers' instructions.

### 2.4 Dual immunofluorescence to detect complex I and complex II in paraffin spinal cord sections

Sections were deparaffinised, rehydrated and antigens were retrieved as described in the previous section. Nonspecific protein interactions were blocked by incubation with 5% normal goat serum (5% NGS) for 30 minutes. Cocktail of primary antibodies was prepared in the blocking solution: anti-cI-19 at 1:200 and anti-cII-70 at 1:400 and the sections were incubated overnight at 4 °C. Following washes with TBST, the sections were incubated with secondary antibody cocktail: anti-IgG2b-Alexa Fluor 499 (Life Technologies, UK) at 1:200 and anti-IgG1-Alexa Fluor 546 (Life Technologies, UK) at 1:200 for 1.5 hour at room temperature. The sections were washed and incubated with DAPI solution for 10 minutes. Autofluorescence was quenched by either immersing the slides in saturated Sudan Black in 70% ethanol for 15 minutes or 0.1% sodium borohydrite in TBS. The sections were mounted in Vectashield permanent mounting medium (Vector Laboratories, UK). Images were collected using Axiovision fluorescent microscope (Carl Zeiss, Germany).

### 2.5 Immunohistochemistry to detect components of complex I and complex II in frozen spinal cord sections

Frozen blocks of lumbar spinal cords were obtained from Newcastle Brain Tissue Resource and London Multiple Sclerosis Society Tissue Bank. Cryosections of 10 μm thickness were collected on glass slides (Superfrost Plus, Thermo Fisher) and 20 μm thick cryosections were collected on polyethylene naphthalate membrane slides (Carl Zeiss, Germany) for subsequent laser capture procedure and molecular analysis of the dissected single cells. The sections were fixed in 4% PFA for 2 minutes, washed in TBST, permeablized in gradient of methanol (70% → 95% + H_2_O_2_ → 100% and 100% → 95% + H_2_O_2_ → 70%) with 10 minutes incubation in each solution. The sections were washed in TBST and protein block (5% NGS for dual immunofluorescence or 5% BSA for immunohistochemistry (IHC) using polymer detection system) was applied to cover each section. The sections were incubated for 30 minutes at room temperature. Subsequent steps were performed as described in the previous sections.

### 2.6 Cell counts using stereology microscope

Paraffin embedded lumbar spinal cord sections were stained for cI-19 or cI-20 using Vector SG chromogen as described previously. Two or 3 (depending on lumbar region) nonserial sections from each case were used for counting using a stereological workstation with a modified light microscope (Olympus, Japan), motorized stage, CCD color camera and stereology software (Stereo Investigator, MBF Bioscience, USA). On average, 150 motor neurons were counted for each case. Entire ventral horn regions were delineated and all motor neuron cell bodies were included in the analysis. IHC staining was evaluated by combined densitometry and visual inspection and the neurons were allocated to one of the 3 categories based on the intensity of signal—“+” normal, “±“ reduced, and “−“ deficient. Counting and categorization of neurons was carried out by one researcher but then verified by another person. The samples were blinded in terms of clinical details and age of the individuals.

### 2.7 Preparation of the material for laser microdissection

Polyethylene naphthalate membrane slides were immunofluorescently dual labeled for cI-19 and cII-70 and entire ventral horn areas were visualized using Axioimager. High-resolution sequential images were collected at 20× magnification and subsequently stitched into a map, which was used to depict complex I deficient motor neurons. The remaining slides were labeled for complex I using Vector SG chromogen. Entire ventral horn regions were visualized in bright field with Axioimager and high resolution maps were created. The same sections were subsequently stained with Eosin to ensure that no complex I-deficient motor neuron was missed because of poor tissue morphology. Complex I-normal, reduced, and negative motor neuron were identified and cut out of the slides using PALM Micro Beam system (Carl Zeiss, Germany). Individual laser microdissected neurons were captured directly into 10 μL of lysis buffer (Tris HCl, 1% Tween, Proteinase K) and incubated on ice as previously described (Murphy et al., 2008).

### 2.8 Analysis of mtDNA copy numbers and deletion level

Multiplex real-time PCR assay was used to detect expression level of 2 genes: MTND1 and MTND4 simultaneously in one sample, as described previously (Krishnan et al., 2007). Briefly, lysates of individual laser dissected motor neurons were used for real-time PCR reaction and each cell was run in triplicate. To assess copy number of mtDNA a standard curve was generated by creating serially diluted template DNA of a known size and concentration. Copy number was calculated per unit area of a dissected neuron. Copy numbers were compared only between samples run in the same plate on the same day to ensure reliability of data. mtDNA deletion analysis was carried out using ΔΔCT method where relative difference in expression of MTND1 and MTND4 gave a percentage value of deleted species versus wild type.

### 2.9 Statistical analysis

For assessing statistical significance, parametric tests were used where the data conformed to assumptions of normality. In all other cases nonparametric tests were used. Statistical significance was determined at *p*-value < 0.05.

## 3 Results

### 3.1 COX-deficient motor neurons are uncommon in aged human spinal cords

**Fig. 1:**
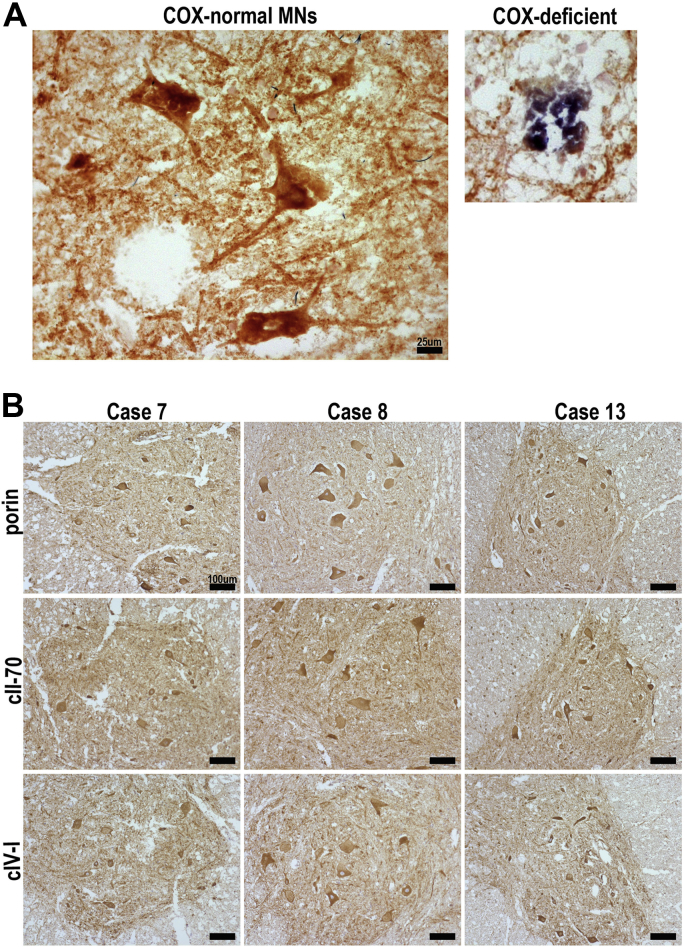
Spinal cord tissue from 9 cases was analyzed for COX and/or SDH activity and protein expression of mitochondrial respiratory complexes. A representative image of respiratory-normal motor neurons in a ventral horn (brown) and a respiratory-deficient motor neuron (blue) (A). Expression of porin (mitochondrial outer membrane protein), complex II subunit 70 kDa (cII-70), and complex IV subunit I (COX-I) of mitochondrial respiratory chain were analyzed in 12 cases using IHC. Typical results are represented by 3 cases (case 7, 8, and 13) (B). Abbreviations: COX, cytochrome c oxidase; IHC, immunohistochemistry; SDH, succinate dehydrogenase. Scale bars measure 25μm (A) and 100μm (B).

To investigate potential mitochondrial involvement in motor neuron loss with age we assessed mitochondrial respiratory capacity by histochemical analysis of COX and SDH activity. We studied a total of 342 motor neurons from 12 different cases and only 1 COX-deficient motor neuron was identified (Fig. 1A). IHC to detect selected mitochondrial proteins showed that cII-70 and COX-I were present in motor neurons of lumbar spinal cord from all 12 cases (Fig. 1B). To assess mitochondrial mass we studied the expression of porin (a mitochondrial outer membrane protein) and there was a comparable expression in all samples (Fig. 1B).

### 3.2 Complex I of mitochondrial respiratory chain is impaired in aging motor neurons

**Fig. 2:**
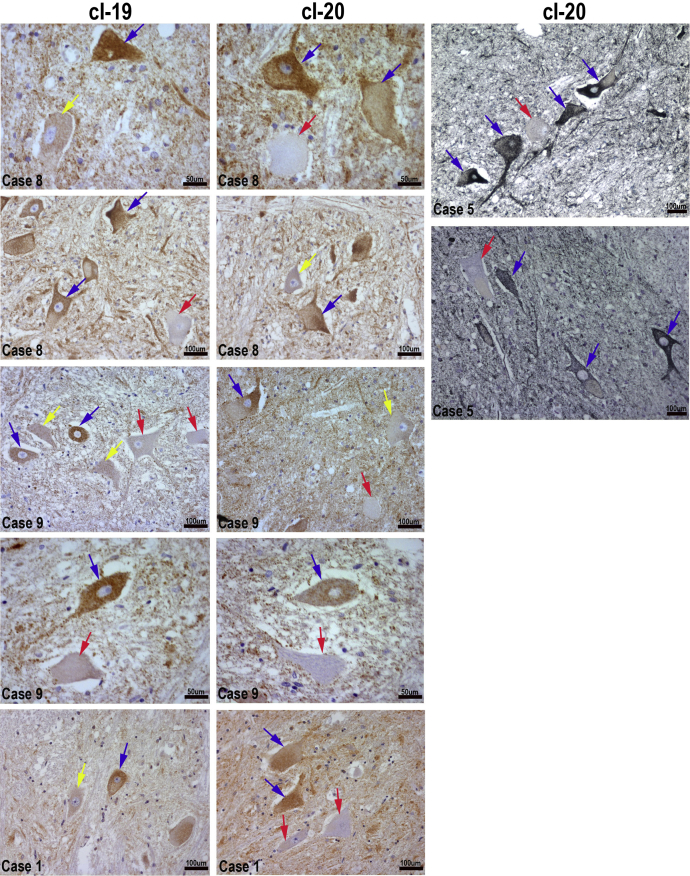
Complex I protein expression detected using antibodies to 2 subunits: 19 kDa (cI-19) and 20 kDa (cI-20) in paraffin-embedded lumbar spinal cord tissue sections from elderly individuals. Signal was developed using DAB (brown) or Vector SG (black), nuclei were counterstained with hematoxylin (blue). Representative images obtained from 4 cases are shown here (cases: 1, 5, 8, and 9). Arrows indicate motor neuron cell bodies: complex I-deficient (red), complex I-reduced (yellow), and complex I-normal (blue). Uneven staining reflects lipofuscin accumulation in motor neurons. Scale bars measure 50µm or 100μm.

**Fig. 3:**
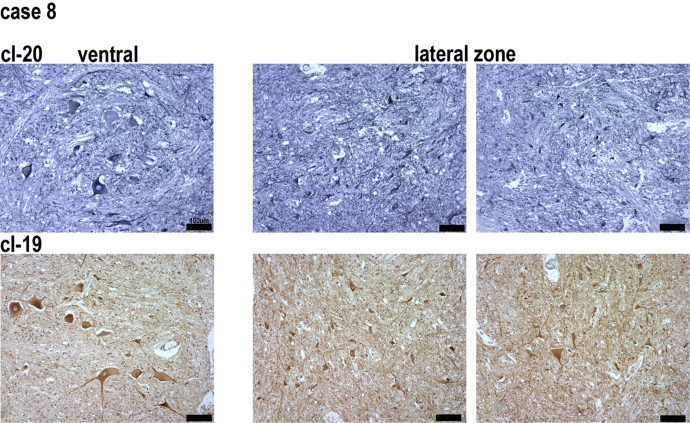
Complex I IHC in ventral and lateral areas of aged spinal cord obtained from case 8. cI-20 were visualized with Vector SG chromagen and cI-19 with DAB. All neuronal cell bodies in the lateral area express comparable levels of complex I proteins. Scale bars signify 100 μm. Abbreviation: IHC, immunohistochemistry. Scale bars signify 100μm.

IHC for 2 components of mitochondrial respiratory chain complex I: cI-19 and cI-20 highlighted a population of motor neurons with low levels or complete lack of immunoreactivity. On the basis of complex I expression, we categorized motor neurons into 3 groups: complex I-normal (+), complex I-reduced (±), and complex I-deficient (−) (Fig. 2). Complex I deficiency was restricted solely to motor neurons and no other cell type within spinal cord tissue demonstrated similar phenotype (Fig. 3).

### 3.3 Quantification of complex I deficiency

**Fig. 4:**
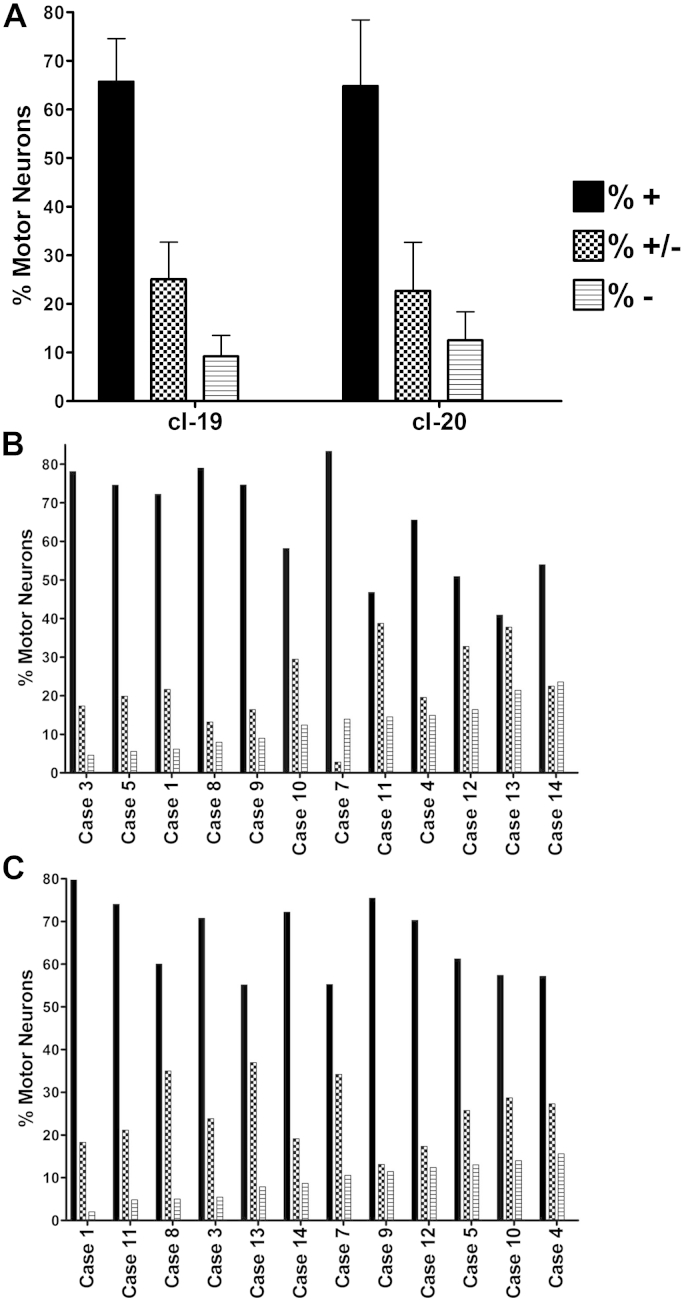
Protein expression levels of cI-19 and cI-20 (mean ± SD) from 12 elderly cases. Two to 3 sections per case were used for analysis and motor neurons were counted in both ventral horn areas. A scale developed based on visual scoring and densitometry allowed categorization of motor neurons into 3 groups: “+” complex I-normal (filled bars), “±“ complex I-reduced (checked bars), “−“ complex I-deficient (striped bars) (A). The proportion of motor neurons in each of the 3 groups in individual cases based on cI-20 (B) and cI-19 immunohistochemistry (C). Abbreviation: SD, standard deviation.

The degree of complex I loss was quantified in 12 elderly individuals. On average 9.2 ± 4.2% and 12.5 ± 6.1% of motor neurons were completely deficient for cI-19 and cI-20, respectively (Fig. 4A). Complex I-reduced cells were more abundant: 25.1 ± 7.6% (cI-19) and 22.7 ± 10.5% (cI-20) (Fig. 4A). The proportion of motor neurons in each of the 3 groups differed between individuals (Fig. 4B and C). Although the severity of respiratory deficiency did not correlate with the age in this small series, all the subjects had a degree of complex I deficiency (Table 1).

### 3.4 Presence of complex I-deficient motor neurons in elderly spinal cords confirmed by immunofluorescence

**Fig. 5:**
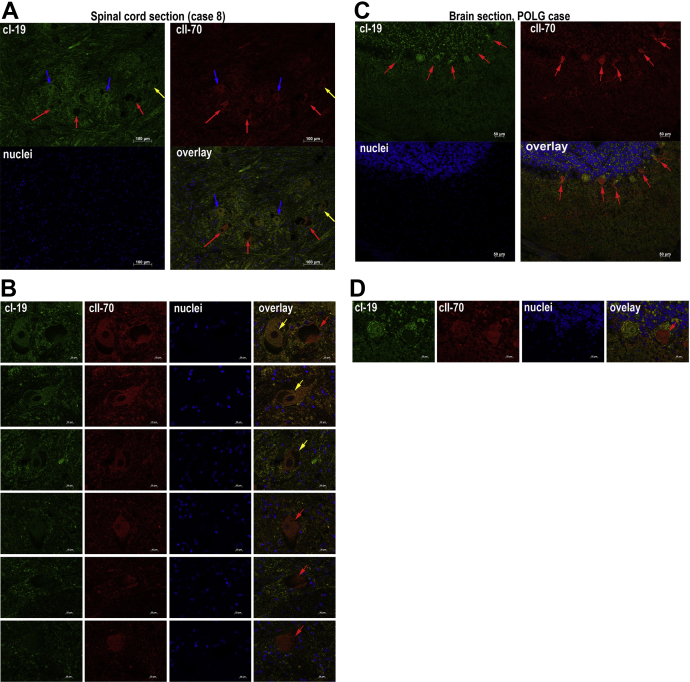
Complex I deficiency in aged spinal cord sections assessed with dual immunofluorescence. cI-19 stained green (FITC) and subunit cII-70 stained red (TRITC), nuclei visualized with DAPI. Paraffin embedded spinal cord tissue section from an elderly subject (case 8) (10× magnification) (A). Single motor neurons from the same case (40× magnification) (B). Paraffin embedded brain section (cerebellum) from a patient with a POLG mutation, where severe complex I deficiency has been previously described (10× magnification) (C). Single complex I-deficient motor neurons from the same brain section (40× magnification) (D). Red arrows highlight complex I-deficient neurons (−), blue: complex I-normal (+) and yellow: complex I-reduced motor neurons (±). Abbreviation: POLG, polymerase γ. Scale bars measure: 100 μm (A), 20 μm (B, D), and 50 µm (C).

To confirm the complex I deficiency we also performed dual immunofluorescent labeling with antibodies to complex I (cI-19) and complex II (cII-70) in the aged lumbar spinal cord tissue (Fig. 5). A mixture of complex I-deficient and/or complex I-reduced motor neurons together with complex I-normal motor neurons were detected in all aged ventral horns analyzed as shown in representative images obtained from case 8 (Fig. 5A and B). These results were compared with a spinal cord section from a patient with a known mitochondrial defect (Fig. 5C and D).

### 3.5 Complex I-deficient motor neurons are not seen early in life

**Fig. 6:**
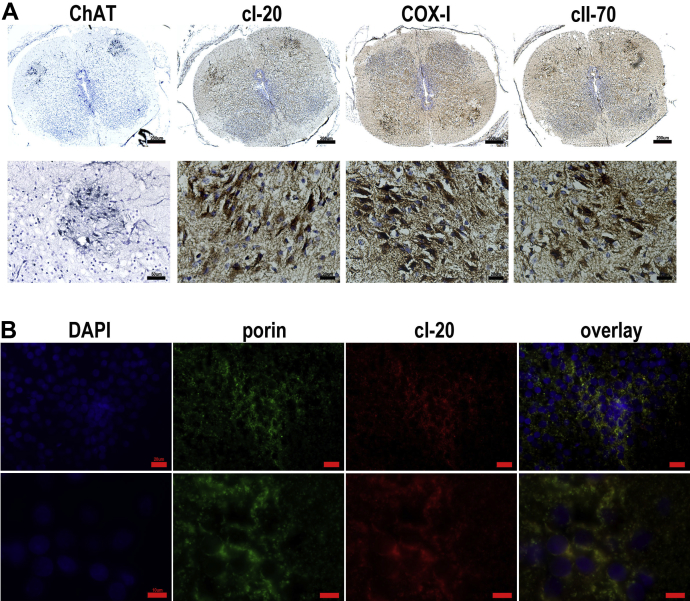
Expression of complex I, II, and IV in fetal spinal cords. Motor neuron population depicted in 9wpc spinal cord section by ChAT labeling using Vector SG and expression of cI-20, COX-I, and cII-70 visualized using DAB (A). A different 9wpc fetal case dual immunolabelled for cI-20 and porin (B). In A scale bars measure 200μm (top panel) or 20 μm (bottom panel except for the image of ChAT: 50μm). In B scale bars signify 20 μm (top panel) or 10 μm (bottom panel).

Human fetal spinal cords obtained from embryos of 9–11 weeks post conception were subjected to IHC and immunofluorescence to assess mitochondrial density as well as expression of selected mitochondrial respiratory chain components. Motor neurons were identified in lumbar spinal cord sections with an antibody against ChAT and consecutive sections were labeled with cI-20, COX-I, and cII-70 using chromogens or immunofluorescence (Fig. 6A and B). No complex I-deficient cells were detected.

### 3.6 Mitochondrial DNA copy number but not deletion heteroplasmy level, correlates with complex I deficiency

**Fig. 7:**
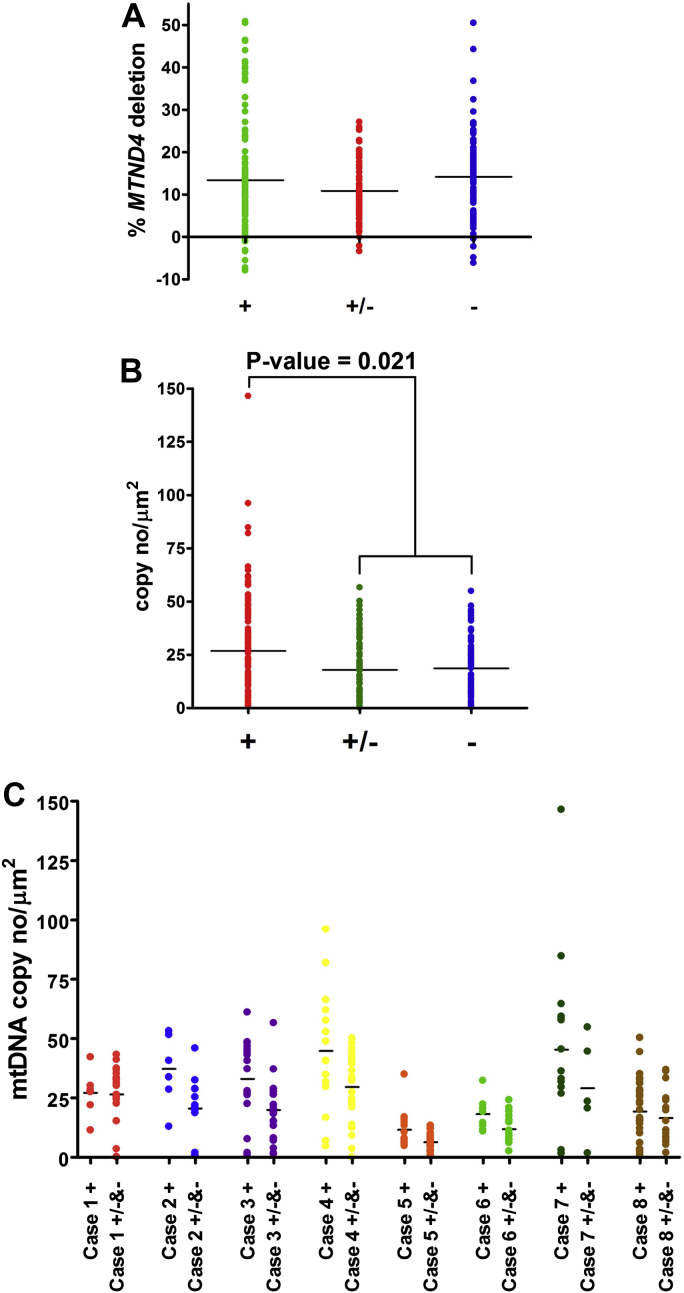
mtDNA deletion levels and mtDNA copy numbers in single motor neurons from 8 cases. Complex I-normal (+), complex I-reduced (±), and complex I-deficient (−) cells were compared. Each dot represents percentage of deletion in mtDNA from a single motor neuron (A). mtDNA copy numbers from complex I-normal (+) motor neurons were compared with complex I-reduced (±) and deficient (−) motor neurons. The difference was statistically significant (*p*-value = 0.021) (Wilcoxon rank-sum test). Each dot represents copy number reading for a single neuron normalized to an area unit (μm^2^) (B). Copy number of mtDNA in single motor neurons showing different complex I status from 8 elderly cases. Copy numbers of complex I-normal were compared with complex I-reduced and complex I-deficient motor neurons (C). Abbreviation: mtDNA, mitochondrial DNA.

Single motor neurons from 8 elderly cases were assessed using real-time multiplex assay to measure deletion load and copy number of mtDNA molecules (Fig. 7). Between 7 and 36 motor neurons were analyzed for each category from each case. In total data were obtained from 119 complex I-normal motor neurons, 62 complex I-reduced motor neurons, and 101 complex I-deficient motor neurons. Although low levels (up to 50%) of mtDNA deletions were detected in motor neurons from all 8 cases examined, there was no statistical difference between complex I-normal (+) complex I-reduced (±), and complex I-deficient (−) subpopulations of motor neurons (Fig. 7A).

Mitochondrial genome copy number analysis showed differences between the defined motor neuron groups (Fig. 7B). All 8 cases combined showed decreased copy numbers in motor neurons with reduced or absent complex I expression. Median copy number normalized to a unit area (μm^2^) was 26.9 for complex I-normal cells, in comparison to 17.9 and 18.6 for complex I-reduced and complex I-deficient, respectively. The difference was statistically significant for complex I-normal versus complex I-reduced and complex I-normal versus complex I-deficient motor neurons (*p*-value = 0.021 for + versus ± or − using Wilcoxon rank-sum test) (Fig. 5B). This indicates that a group of cells demonstrating reduced expression of complex I and a group of complex I-deficient cells held very similar mtDNA density, which was lower than in cells with preserved complex I. Copy number in individual cases are plotted in Fig. 7C.

### 3.7 Motor neuron size alteration

**Fig. 8:**
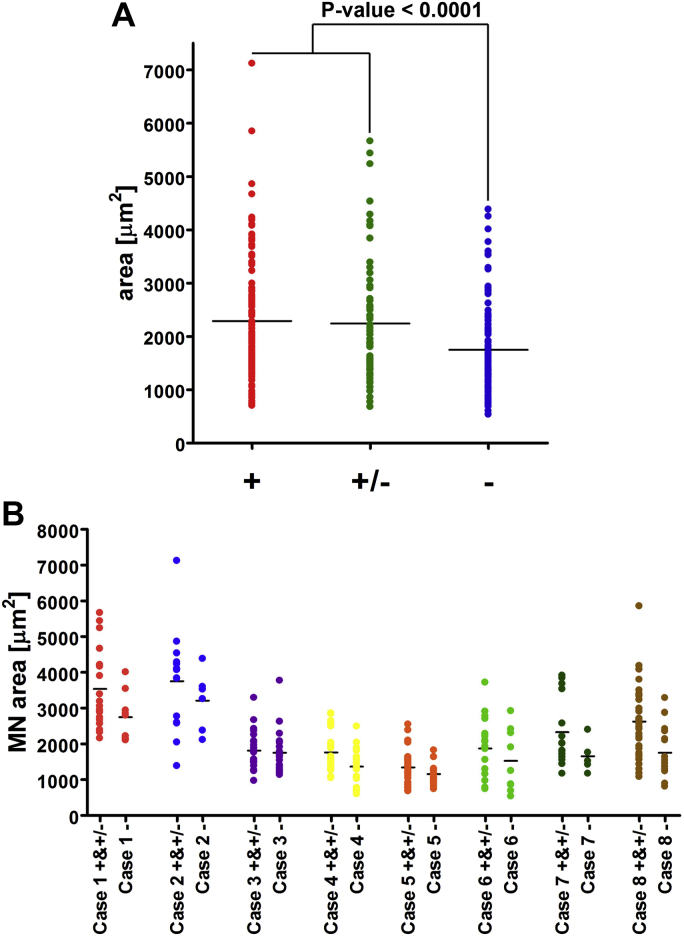
Size of individual motor neurons represented as area from 8 elderly cases. Each dot shows a measurement from a single cell. Differences in area were statistically significant with *p*-value < 0.0001 (A). A comparison between complex I-normal (+) and complex I-reduced (±) versus complex I-deficient (−) for individual cases (B).

We measured the area of motor neuron cell bodies belonging to one of the 3 categories: complex I-normal, complex I-reduced, and complex I-deficient. Groups of complex I-normal and complex I-reduced comprised motor neurons of almost identical size with mean area values of 2289 μm^2^ (±1110) and 2245 μm^2^ (±1191), respectively. Complex I-deficient motor neurons, on the other hand, were significantly smaller than both the aforementioned groups and measured on average 1748 μm^2^ (±848) (*p*-value < 0.0001 versus complex I-normal, *p*-value = 0.0003 versus complex I-reduced and [Fig. 8A]). The size of motor neurons differed significantly between the individuals (Fig. 8B).

## 4 Discussion

Sarcopenia is an aging phenomenon with loss of both muscle mass and strength. The etiology is likely to be multifactorial but motor neuron loss is believed to play an important role. We report that a significant portion of motor neurons in elderly individuals harbor mitochondrial complex I deficiency. The average level of complex I deficiency was around 10% in 12 individuals aged 70–99 years. We also identified motor neurons with markedly reduced complex I expression, which constituted about 25% of the total spinal motor neuron pool. We detected only 1 COX-deficient motor neuron using histochemistry for COX and SDH enzymatic activity and thus COX deficiency, unlike complex I deficiency, is rare. The mitochondrial mass was preserved as both complex II and porin remained uniformly expressed in all motor neurons. It is important to highlight the fact that there is no evidence for different rates of decline of mitochondrial complexes dependent on postmortem delay (Harish et al., 2013). There was no correlation between the length of the postmortem delay and the proportion of complex I-deficient motor neurons in our cohort (Table 1, Fig. 4). Molecular analysis carried out on single complex I-deficient and complex I-reduced motor neurons showed that these cells contained significantly fewer mtDNA copies than healthy motor neurons. Finally, we observed that the complex I-deficient neurons were significantly smaller than both the complex I-normal and complex I-reduced cells.

Our observation of complex I deficiency in aged motor neurons is intriguing, as is its link with mtDNA depletion. There is good evidence that in the presence of an mtDNA defect complex I is the first respiratory chain complex affected (Kirby et al., 1999). In postmortem studies of patients with infantile-onset spinocerebral ataxia, a neurodegenerative disease caused by mutations in the mitochondrial helicase Twinkle, the cerebrum, and the cerebellum showed severe depletion of mtDNA. As a consequence of this mtDNA depletion, most of the neurons in the cerebellum and the frontal cortex had reduced or completely absent immunoreactivity against 3 complex I subunits (Hakonen et al., 2008). Interestingly, complex II and IV immunoreactivity were unaltered in both brain regions. These observations seem to closely resemble pattern of mitochondrial abnormality reported in motor neurons in this study, although the decrease we observed in mtDNA copy number was not as marked. In a mouse model with tissue specific knockout of mitochondrial transcription factor A (Ekstrand and Larsson, 2002), mtDNA depletion in cortical neurons leads to downregulation of mitochondrial transcripts, including complex I, and causes widespread neurodegeneration (Silva and Larsson, 2002).

Other studies exploring the mitochondrial respiratory chain protein expression in mitochondrial disease have also highlighted that complex I deficiency is often the most prominent defect seen. In a series of patients with different mitochondrial defects and ataxia, a high proportion of cerebellar neurons had complex I deficiency and only a small fraction of these neurons demonstrated COX down-regulation. These studies also showed that there is a progression of respiratory chain deficiency in the patients and COX down-regulation appears later in the course of disease (Lax et al., 2012). Deficiency of COX was strongly correlated with cell loss, implying that COX is essential to neuronal viability. One of the reasons we detected only very low levels of COX deficiency may be that motor neurons are particularly sensitive to the defect of this complex and thus lost before they can be detected. If this is the case then the complex I deficiency we observe may be part of a progressive process which inevitably leads to the neuronal loss known to occur with aging. Data obtained from a number of complex I knockout animal models provide further evidence supporting that complex I deficiency leads to a serious pathology. A mouse model of complex I deficiency created by knocking out NDUFS4, a nuclear encoded complex I subunit, presented severe neurodegeneration (Leigh syndrome-like phenotype) and death within the first 7 weeks (Kruse et al., 2008). The same phenotype developed in mice where NDUFS4 knockout was restricted to neurons and glia (Quintana et al., 2010). In the wobbler mouse, which serves as a model of amyotrophic lateral sclerosis, an isolated complex I inhibition appeared in spinal motor neurons at an early stage and correlated with the disease onset (Dave et al., 2003; Jung et al., 2002).

Aging human postmitotic tissues have been shown to accumulate clonally expanded mtDNA deletions causing respiratory deficiency (Greaves and Turnbull, 2009). In this study, however, we did not detect significantly increased mtDNA deletions in the complex I-deficient cells suggesting that either mtDNA deletions are not the cause of pathology in these motor neurons or they had not accumulated to high enough levels. We did observe in both the complex I-reduced and complete complex I-deficient motor neurons mtDNA depletion as compared with cells with normal complex I levels. mtDNA depletion could result from a variety of factors including nuclear genetic defects, limitation of nucleosides or even be secondary to relative inactivity of the neuron. Unfortunately in postmortem tissue it is impossible to identify the underlying mechanism.

Another important question relates to the consequence of the complex I defect on motor neuron function or survival. We observed motor neuron soma size reduction in the complex I-deficient neurons. Interestingly, this was only seen in the complex I deficient neurons, suggesting that the progressive loss of complex I level does have a direct effect on neuron function. We cannot exclude a possibility that smaller motor neurons are more susceptible to acquiring complex I damage although we believe that is less likely scenario. We also know from patients with mitochondrial disease that complex I deficiency can certainly lead to severe neuropathological defects. The most common condition often associated with isolated complex I deficiency is Leigh syndrome, a progressive neurodegenerative condition (Fassone and Rahman, 2012; Pagliarini et al., 2008; Smeitink et al., 2004). Therefore, the defect we have observed in motor neurons is likely to have an effect on neuronal function and/or viability.

In conclusion, we have identified complex I deficiency in motor neurons in aged human spinal cords. We believe that respiratory chain deficiency in motor neurons may be an important mechanism, which leads to the motor neuron dysfunction and loss in sarcopenia. Understanding both the causes and consequences of this deficiency is important and may give new insights into future therapeutic interventions to treat or prevent sarcopenia.

## Disclosure statement

The authors disclose no conflicts of interest including any financial, personal, or other relationships with other people or organizations, which could inappropriately influence their work.

## Figures and Tables

**Table 1:** Individual cases included in the study. All the cases were deprived on any muscle conditions hence treated as healthy controls in terms of sarcopenia

Elderly individuals studied				
Individual	Age (y)	Gender	PM (h)	Cause of death
Case 1	74	Male	21	MI, IHD
Case 2	87	Male	21	Heart disease
Case 3	77	Male	22	Cardio pulmonary degeneration
Case 4	68	Male	10	Colon cancer
Case 5	85	Male	23	Heart failure
Case 6	87	Female	72	Hemopericardium because of ruptured MI
Case 7	96	Female	95	Acute subdural hematoma 1a AF
Case 8	86	Female	75	Old age
Case 9	81	Male	22	Left ventricular failure because of MI
Case 10	74	Female	45	Pulmonary demyelinating polyneuropathy
Case 11	73	Male	24	Left ventricular failure, coronary artery bypass graft thrombosis
Case 12	99	Male	111	Old age
Case 13	74	Female	67	Lung cancer
Case 14	75	Female	31	Left ventricular failure because of MI

Key: IHD, ischemic heart disease; MI, myocardial infraction; PM, post mortem delay.
